# Controlled clustering enhances PDX1 and NKX6.1 expression in pancreatic endoderm cells derived from pluripotent stem cells

**DOI:** 10.1038/s41598-020-57787-0

**Published:** 2020-01-27

**Authors:** Raymond Tran, Christopher Moraes, Corinne A. Hoesli

**Affiliations:** 1 Department of Chemical Engineering, McGill University, 3610 rue University, Montreal, QC Canada; 2 Department of Biomedical Engineering, McGill University, 3775 rue University, Montreal, QC Canada; 3 Rosalind and Morris Goodman Cancer Research Center, McGill University, Montreal, QC Canada

**Keywords:** Induced pluripotent stem cells, Biomedical engineering, Surface patterning

## Abstract

Pluripotent stem cell (PSC)-derived insulin-producing cells are a promising cell source for diabetes cellular therapy. However, the efficiency of the multi-step process required to differentiate PSCs towards pancreatic beta cells is variable between cell lines, batches and even within cultures. In adherent pancreatic differentiation protocols, we observed spontaneous local clustering of cells expressing elevated nuclear expression of pancreatic endocrine transcription factors, PDX1 and NKX6.1. Since aggregation has previously been shown to promote downstream differentiation, this local clustering may contribute to the variability in differentiation efficiencies observed within and between cultures. We therefore hypothesized that controlling and directing the spontaneous clustering process would lead to more efficient and consistent induction of pancreatic endocrine fate. Micropatterning cells in adherent microwells prompted clustering, local cell density increases, and increased nuclear accumulation of PDX1 and NKX6.1. Improved differentiation profiles were associated with distinct filamentous actin architectures, suggesting a previously overlooked role for cell-driven morphogenetic changes in supporting pancreatic differentiation. This work demonstrates that confined differentiation in cell-adhesive micropatterns may provide a facile, scalable, and more reproducible manufacturing route to drive morphogenesis and produce well-differentiated pancreatic cell clusters.

## Introduction

Type 1 diabetes is caused by the autoimmune destruction of the insulin-producing beta cells found in the islets of Langerhans in the pancreas. Islet transplantation is a promising long-term cell-based therapy that provides insulin independence in more than 85% of recipients for at least 1 year^1,2^. Access to islet transplantation remains limited by donor islet availability. Insulin-secreting cells derived from pluripotent stem cells (PSCs) are a possible source for these therapies, provided that robust differentiation protocols can be developed^3–6^. The efficiency of mature beta cell production from PSCs remains limited and variable between cell lines, protocols, and even batches within the same research group^3,7,8^. Although more mature beta cell clusters can be obtained via cell sorting and controlled aggregation, these additional processing steps may significantly reduce overall yields and are undesirable to maximize beta cell production^9^. While early steps in the differentiation process are well-established and reasonably efficient, the successful production of pancreatic endoderm (PE) cells from pancreatic foregut (PF) cells is less consistent, and incomplete differentiation at this stage is expected to affect downstream specification^10^. Strategies to improve differentiation efficiency and PE cell yield from PF cells could substantially improve the robustness and overall efficiency of beta cell production from PSC sources.

PDX1 and NKX6.1 are the earliest markers of pancreatic and beta cell commitment, respectively^11–13^, and play a critical role in pancreatic development towards functional insulin secretion capability^14–16^. Overexpression of PDX1 promotes differentiation towards insulin-expressing cells in pancreatic differentiation of mouse and human embryonic stem cells (hESCs)^17,18^. Nuclear translocation of PDX1 through phosphorylation is required for activation and binding to the insulin promoter^19–21^ and other PDX1-binding DNA motifs^22–24^. NKX6.1 represses the formation of multihormonal endocrine cells^25^ and higher NKX6.1 expression correlates with accelerated maturation of hESC-derived PE cells into insulin-expressing cells after engraftment in diabetic mice^26^. Functionally, PDX1 and NKX6.1 also contribute to mature beta cells survival and synthesis of insulin^11,16,27^. High yields of PDX1^+^/NKX6.1^+^ PE cells can be achieved by implementing a multicellular aggregation step^4,5,8^. Current differentiation protocols involve cell release from the surface and then aggregate formation. These aggregates are typically heterogenous which may explain batch variability observed in insulin-producing cell yield, maturity, and purity. More advanced techniques such as microfluidic methods^28^ or cell-repellent microwells can result in homogenous structures, but these are challenging to scale up, can require complex equipment and/or multiple manual operation steps which ultimately leads to significant loss of valuable cell material. These challenges all arise because they require cell detachment from adherent substrates prior to further processing and aggregation. Developing techniques that allow the formation of aggregates while maintaining adhesion might be a viable strategy to avoid these issues.

In this work, we propose that culture in adhesive micropatterns can be applied to direct and control cell clustering for efficient pancreatic differentiation in a scalable manner. Cells grown on small adhesive 2D micropatterned surfaces have previously been shown to form 3D aggregates of well-defined and uniform sizes when released^29,30^. This suggests that micropatterned surfaces mechanically prime cells to form clusters, which may in itself be sufficient to improve PE cell yields. In this work, we culture adherent induced PSC (iPSC)-derived PF cells on micropatterned surfaces and demonstrate that sufficiently small patterns prompt clustering into multilayered structures during the PE transition, while cells are retained on the adherent surfaces. Cell-adhesive microwells induced higher levels of PDX1 and NKX6.1 nuclear transcription factor accumulation in the overall cell population, and this increase was associated with the clustering phenotype in which multilayer tissues are formed. Overall, this system maintains the simplicity and ease of handling possible with simple adherent 2D culture systems, while enhancing differentiation efficiency and may hence provide a scalable route towards cell therapy manufacturing.

## Results

### Establishing pancreatic differentiation baseline in unconfined monolayer culture

**Figure 1:**
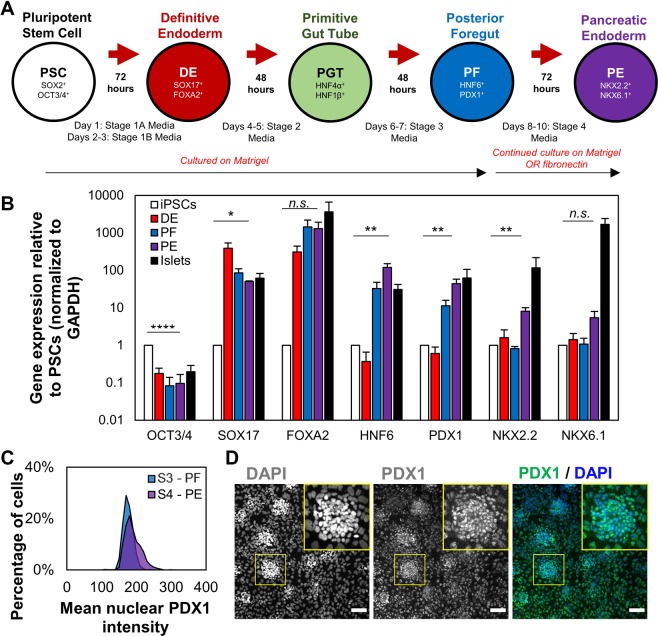
Differentiation of iPSCs into PF cells on tissue culture plastic with timed addition of soluble factors without confinement. (**A**) The stages of development are mimicked by timed media changes and monitored by expression of key transcription factors. (**B**) Expression of key pancreatic transcription factors relative to GAPDH based on qPCR. No significant differences were found between β-actin and GAPDH. Each bar represents the average of three separate differentiations (N = 3). (**C**) Mean nuclear PDX1 intensity at the PF and PE stages of differentiation, showing a small proportion cells with higher PDX1 expression (PDX^high^) emerging at the PE stage. (**D**) In monolayer cultures, roughly circular aggregates of PDX1^high^ cells were observed sporadically in culture. n.sp > 0.05, *p < 0.05, **p < 0.01, ****p < 0.001 for a one-way ANOVA. Full Tukey multiple comparisons post-hoc test results are shown in Table S1. Scale bars represent 100 µm.

To establish a baseline differentiation efficiency for iPSCs in our hands, pancreatic endoderm (PE) cells were produced using an established differentiation protocol in adherent unconfined monolayer culture (Fig. 1A, Table S1)^5^. Commitment from pluripotent states towards pancreatic lineages was confirmed by the downregulation of pluripotency gene, OCT3/4, compared to iPSCs, as well as upregulation of definitive endoderm genes, SOX17 and FOXA2, relative to glyceraldehyde-3-phosphate dehydrogenase (GAPDH). After generation of PF cells, the mRNA levels of pancreatic genes, PDX1 and HNF6 increased to reach expression levels similar to healthy adult human islets, indicating commitment towards the pancreatic lineage during the differentiation process (Fig. 1B). As expected, NKX6.1 mRNA levels were not increased after induction of the PF stage. Contrary to other reports^3–5^, NKX6.1 mRNA levels were also not upregulated in PE cells generated from standard unconfined cultures compared to undifferentiated iPSCs in our hands^11^. The nuclear fluorescent intensity of immunostained cells indicated a measurable but highly variable nuclear expression of PDX1 in >90% of the cell population (Fig. 1C).

Since only a small fraction of cells demonstrated increased nuclear PDX1 concentration at the PE stage, we asked whether there were any spatial patterns to the variable differentiation efficiency. When we analyzed the distribution of brightly labelled PDX1 cells (denoted PDX1^high^) in monolayer culture after the transition from PF to PE, these differentiated cells were clustered together (Figs. 1D, S1), while the majority of cells expressed lower levels of nuclear PDX1. The size and position of these clusters appeared to be random, suggesting a stochastically-driven differentiation process that may lead to poor differentiation outcomes downstream. PDX1^high^ clusters were circular in size and about 150 µm in diameter. Based on these observations, we then asked whether directing and controlling the formation of adherent cell clusters could improve pancreatic differentiation efficiency in the overall cell population and developed a micropatterning system to test this hypothesis.

### Micropatterned adhesive islands prompt PF cell clustering and localized multilayer tissue formation

**Figure 2:**
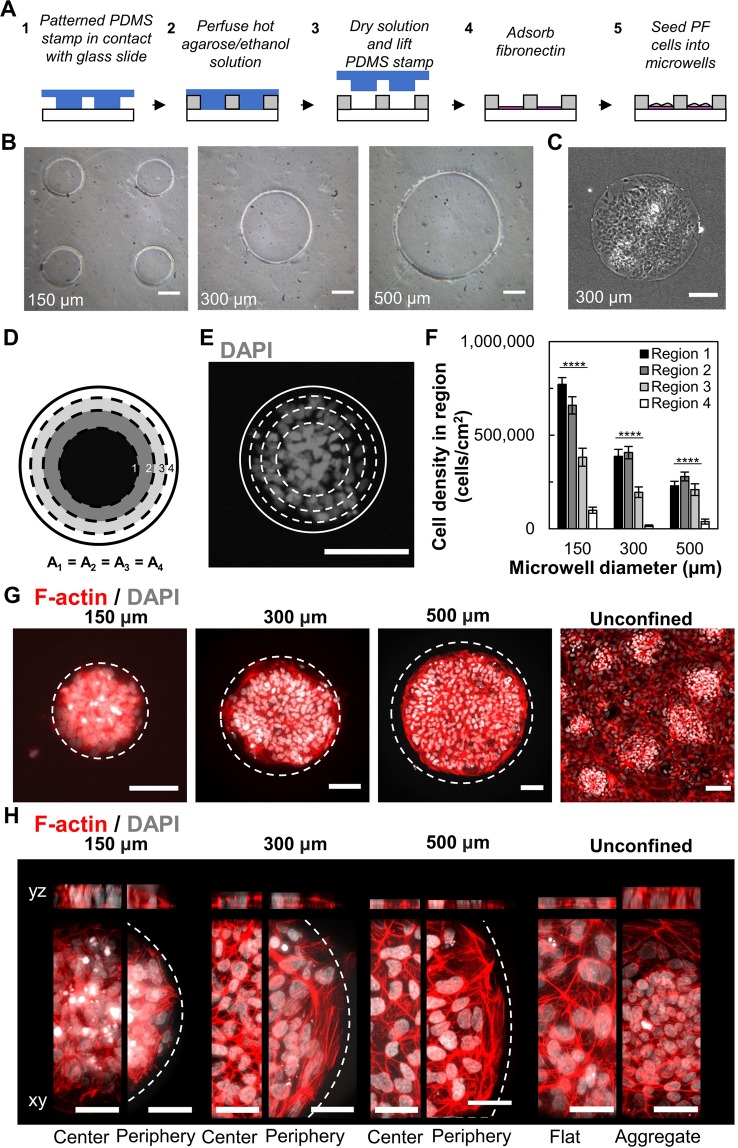
Confined culture of iPSC-derived PF cells in micropatterns promotes clustering of cells in predictable patterns. (**A**) Process flow to microfabricate agarose microwells to spatially confine iPSC-derived PF cells. (**B**) Agarose microwells of 150 µm, 300 µm and 500 µm diameters were successfully fabricated with high between-well consistency in diameter. (**C**) Microwells facilitate confined cell clustering within 72 hours. (**D**) Areas of 4 concentric circles used for spatial analysis of cell density. (**E**) Nuclei are concentrated within inner radii of microwells. (**F**) Local cell density in concentric microwell regions for 150 (n = 21), 300 (n = 13), and 500 (n = 9) µm microwells. (**G**) Cell densification occurs in 150 µm microwells similar to aggregates in unconfined culture. (H) Bulged morphology observed after 72 hours in 150 µm microwells but not in 300 µm or 500 µm microwells. Representative images of max intensity projection and 3D reconstructions in yz (top) and xy (bottom) plane. ****p < 0.001 for an one-way ANOVA performed on the iPSC-derived cells. Full Tukey multiple comparisons post-hoc test results are shown in Table S2. Scale bars represent 100 µm (**B,C,E,G**) and 50 μm (**H**).

To facilitate controlled cell clustering, circular cell-adhesive microwells were fabricated to spatially confine PF cells during differentiation in adherent culture, using an agarose-based microwell fabrication process (Fig. 2A)^31^. Microwells with diameters ranging from 150 µm to 500 µm were successfully fabricated (Fig. 2B) using standard soft lithography and replica molding processes, as previously demonstrated by others^32^. The agarose patterning technique was suitable to spatially confine cells for the 72 hours required for the PE culture step (Fig. 2C). Since local cell density is known to impact pancreatic specification^8,33^, we quantified this parameter in controlled cluster cultures. To assess local cell density, each microwell was segmented into 4 concentric circles with equal area followed by nuclei enumeration (Fig. 2D,E). In all microwells, the cell nuclei were primarily concentrated in the 3 innermost regions (Fig. 2F). Furthermore, the overall microwell cell density decreased with increasing diameter, with the 500 µm microwells having the lowest cell density after 72 hours of confined culture. Increasing the initial seeding density did not appear to increase the cell density within 500 µm microwells over the 72 hours of culture (not shown).

To verify that microwell culture promotes controlled clustering into adherent multilayered microtissues, we visualized the 3D F-actin structure using confocal fluorescent microscopy. In the 150 µm microwells (Fig. 2G,H), a 3D multilayered morphology was observed, resembling the clusters arising stochastically in unconfined monolayer cultures. This multilayered morphology was less pronounced in the 300 µm microwells and not present in the 500 µm microwells. Furthermore, at the edges of the 300 µm and 500 µm microwell colonies, the actin filaments appeared concentrated and aligned with the microwell boundary. This suggests that the actin structures under tension at the micropattern boundary act to cluster the cells, and this tension is sufficiently strong in the smallest patterns to promote cell-driven organization into a multilayer tissue. These results demonstrate that sufficiently small 2D micropatterns can be employed with iPSC-derived PF cells to produce adherent multilayered cell clusters in a predictive and controlled manner.

### Controlled clustering of posterior foregut cells enhances pancreatic nuclear transcription factor localization

**Figure 3:**
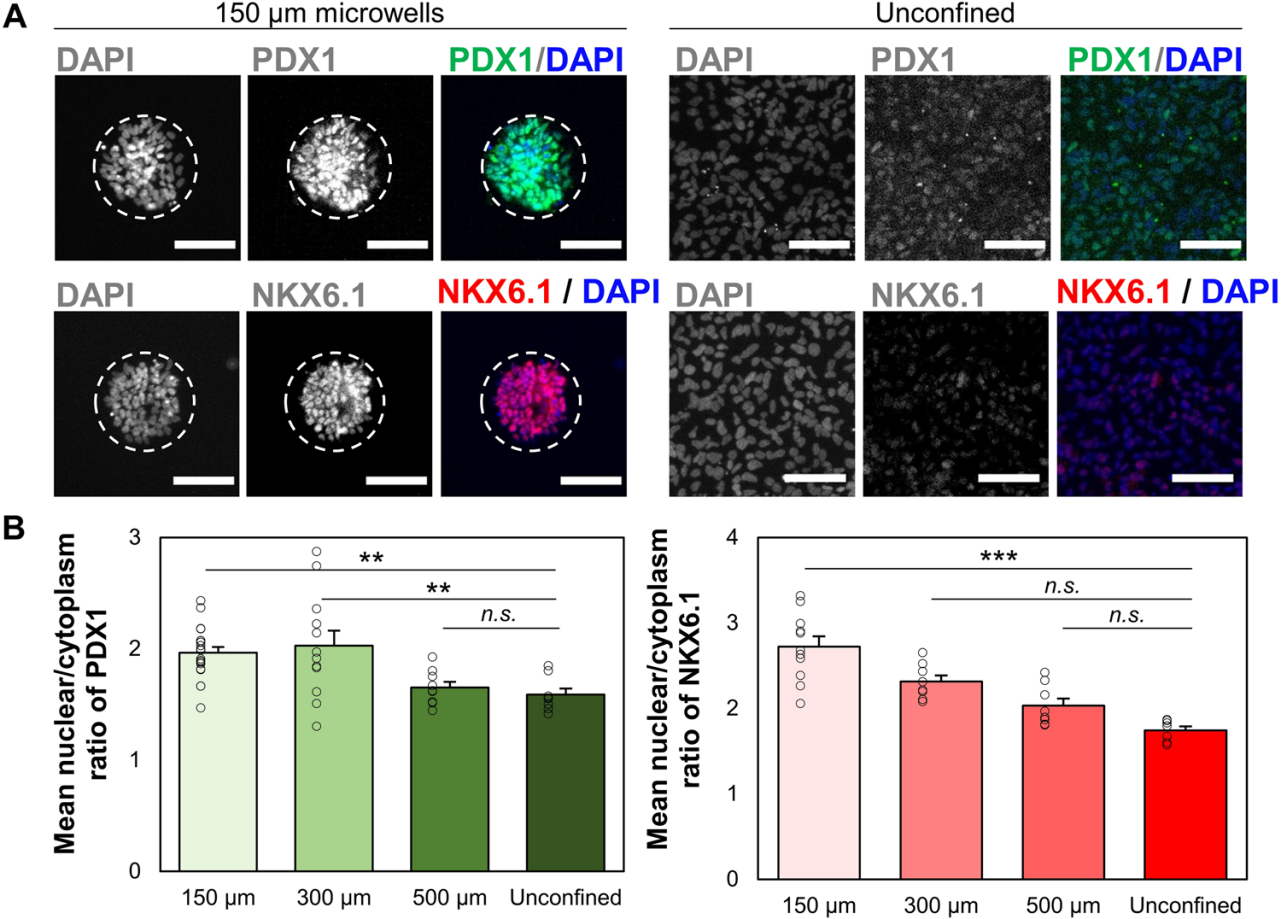
Spatially confined differentiation of PF cells promotes the expression of PE markers. (**A**) Confined culture of iPSC-derived PF cells increases staining intensity of PDX1 and NKX6.1 as shown by immunocytochemistry. Displayed intensity ranges have been matched between confined and unconfined samples to illustrate increased staining intensity (**B**) Mean N:C ratio in PDX1 (n = 21, 13, 9 for 150, 300, and 500 µm microwells) and NKX6.1 (n = 12, 8, 8 for 150, 300, and 500 µm microwells) immunofluorescence increased when presented with sufficient geometric confinement. Each point represents a data point from a single microwell. n.sp > 0.05, **p < 0.01, ***p < 0.005 for a one-way ANOVA with Tukey post-hoc multiple comparisons. Scale bars: 100 µm.

To determine whether microwell culture promoted pancreatic differentiation during the transition from PF to PE, nuclear to cytoplasmic (N:C) expression ratios of PDX1 and NKX6.1 were evaluated via immunostaining based on mean fluorescence intensity (Figs. 3A, S2). N:C ratio was used as a metric since nuclear localization is required for transcriptional activity of these transcription factors and hence for the activation of downstream targets. After 72 hours in PE differentiation medium, cells cultured in the 500 µm microwells did not show any statistically significant increase in the mean N:C ratio for PDX1 or NKX6.1 over the unconfined control. However, the mean N:C ratio of PDX1 was significantly increased in the 150 µm and 300 µm diameter microwells, suggesting that islands with diameters smaller than 500 µm are required to observe significant effects of spatial confinement (Fig. 3B). An overall increase in total nuclear fluorescent accumulation of PDX1 and NKX6.1 was also observed (Fig. S3) and a similar trend in PDX1 and NKX6.1 fluorescent intensity of confined cells was observed (Fig. S4). NKX6.1 expression was significantly increased on the 150 µm diameter microwells only. These results sugests that spatial confinement was sufficient to increase the nuclear amount of PDX1 and thus increase the amount of active transcription factors. Furthermore, differentiation into PE cells was confirmed by co-localization of PDX1 and NKX6.1 in 150 µm microwells (Fig. S5). Hence, 150 µm diameter microwells are sufficiently small to produce clusters that simultaneously upregulate nuclear concentration of both target transcription factors.

**Figure 4:**
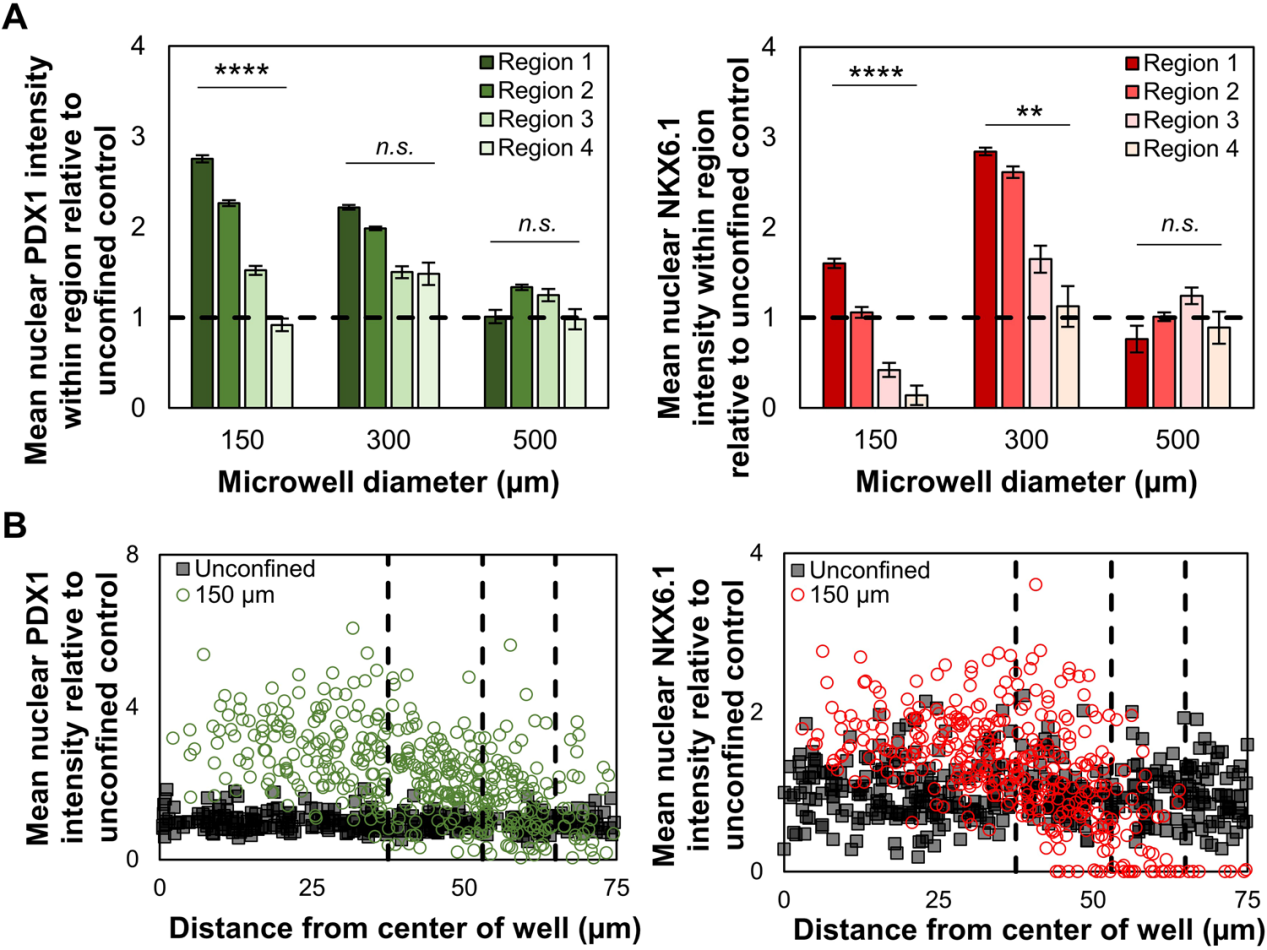
Controlled cluster culture drives spatially dependent patterns of PDX1 and NKX6.1 within the cluster. (**A**) Mean nuclear PDX1 (n = 21, 13, 9 for 150, 300, and 500 µm microwells) and NKX6.1 (n = 12, 8, 8 for 150, 300, and 500 µm microwells) intensity varies region-to-region and shows correlation to local cell densities. (**B**) Confined PF cells show increased PDX1 and NKX6.1 intensity on a single cell level. Dashed lines represent limits of regions defined in Fig. 2D. n.s.p > 0.05, **p < 0.01, ****p < 0.001 for a one-way ANOVA between different regions.

Since cell density affects differentiation outcomes in standard unconfined monolayer cultures, we asked whether local cell density within the clusters correlates with these improved differentiaton profiles. As we have determined the overall trends for N:C ratios of PDX1 and NKX6.1, we studied the nuclear PDX1 and NKX6.1 intensity in 4 concentric regions as defined earlier (Figs. 2D, 4A) within each microwell and found that increased cell density in the center of each individual microwell correlated strongly with increased nuclear PDX1 and NKX6.1 expression. On a single cell basis, the majority of cells cultured within the microwell had increased expression of PDX1 or NKX6.1 (Fig. 4B) with increasing expression towards the centers of the well for NKX6.1. Cells located at the periphery of 150 µm microwells have much lower NKX6.1 fluorescent intensity than the unconfined control. This could be due to the clustering as a result from confined culture, thus resulting in self-organization within the micropatterns. Another potential explanation could be that the decreased cell densities at the periphery inhibit pancreatic differentiation. Furthermore, nuclear NKX6.1 expression was greater in 300 µm microwells than in 150 µm which suggests an ideal microwell size exists maximizes nuclear accumulation of both PDX1 and NKX6.1. Altogether, these data suggest that controlled clustering into appropriately-sized, adherent, multilayered microtissues can significantly impact nuclear expression of transcription factors such as PDX1 and NKX6.1, which are crucial to beta cell commitment.

### Cell clustering and differentiation efficiency is associated with actin-driven morphogenesis

**Figure 5:**
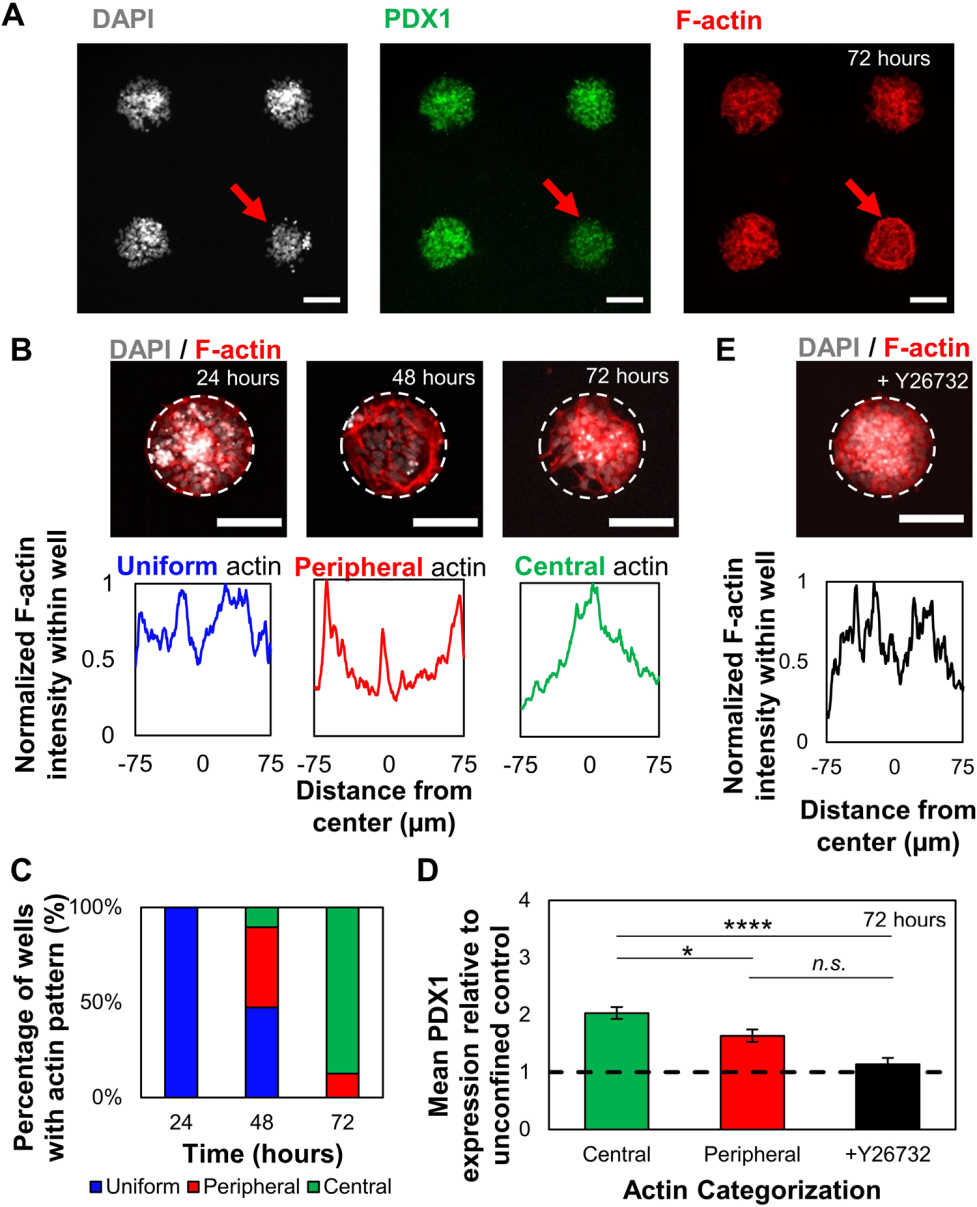
Confined culture promotes temporal changes in F-actin organization during differentiation. (**A**) Micropatterned iPSC-derived PF cell colonies that show visibly lower PDX1 intensity (red arrows) are correlated with distinct actin cytoskeleton structures concentrated at the microwell periphery. (**B**) Characteristic actin structures found throughout 72 hours of differentiation in 150 µm microwells. These structures are grouped based on actin intensity analysis (bottom). The reported intensity profile is obtained from an average of 8 intersecting lines (Fig. S6). (**C**) Proportion of wells with actin structure (classified in (**B**) at 24 (n = 10), 48 (n = 19), and 72 (n = 32) hours. Distribution of actin structures changes throughout differentiation and aggregates towards the center in 150 µm microwells. (**D**) PF colonies in 150 µm microwells with central actin distribution (n = 16) showed increased nuclear PDX1 expression compared to those with peripheral actin distribution (n = 5). Addition of Y-26732 abrogated any increase in nuclear PDX1 expression compared to the unconfined control (n = 12). (**E**) Addition of Y-26732 ROCK inhibitor 24 hours after seeding prevents actin structural reorganization.  *p < 0.05, ****p < 0.001 for the student’s t-test. Scale bars: 100 µm.

Although confined culture in 150 µm microwells consistently increased PDX1 and NKX6.1 nuclear expression compared to unconfined cultures (Fig. 3B), some heterogeneity in actin filament distribution and expression levels were still observed between microwells. We reasoned that this may be due to differences in clustering dynamics between wells. Therefore, we investigated the temporal evolution of the actin cytoskeletal structures associated with formation of these adherent, multilayered microtissues in 150 µm diameter microwells. A correlation was observed between microwells with low mean PDX1 expression and a distinctive actin cytoskeletal architecture where the stain was most intense at the microtissue periphery (Fig. 5A). Conversely, microwells with high mean PDX1 expression showed intense actin staining near the microwell center and a more aggregated morphology, suggesting that the evolving microtissue architecture changes during differentiation.

To determine how such actin patterns arise during the clustering process, we looked at population-based temporal evolution of actin cytoskeletal structures in 150 µm diameter microwells. We observed characteristic actin architectures at 24, 48, and 72 hours of confined differentiation and classified them as either uniform, peripheral, or central based on the predominant actin morphology observed within the microwell (Fig. 5B). Uniform actin filament distribution was observed 24 hours after seeding and culture in the 150 µm microwells. After 48 hours, some microwells developed well-defined F-actin structures concentrated at the microwell periphery. At 72 hours, a large proportion of the microwells had high intensity actin cytoskeletal structures develop in the center of the microwell (Fig. 5C). The progression from ‘uniform’ to ‘peripheral’ to ‘central’ actin architectures suggests that the peripheral actin ring first forms around the colony, and then acts to contract the colony inwards to the microwell center. Furthermore, the large proportion of microwells with ‘central’ actin architectures suggests controlled clustering could be used to significantly reduce variability in otherwise stochastic cultures. We therefore hypothesize that centralized actin architectures are characteristic of wells that have undergone clustering morphogenesis to form multilayered microtissues, while other actin architectures represent earlier stages in the clustering process.

To determine whether the clustering is caused by pancreatic differentiation or a natural consequence of microwell geometry, confined culture was performed in basal medium without added PE differentiation factors. As expected, removing the differentiation factors abrogated the increases in PDX1 expression in both confined and unconfined monolayer culture (Fig. S6A, B). It is important to note that reduced cell density was observed in the unconfined monolayer culture in the absence of differentiation factors (Fig. S6C), suggesting that population-growth driven changes in density may play a role in nuclear PDX1 concentration. Without PE differentiation factors, most microwells were filled with cells, and exhibited uniformly distributed actin architectures after 72 hours (Fig. S6A,B), suggesting that the process of inducing differentiation itself is required for F-actin peripherally focused structures to drive cell clustering.

We then hypothesized that variations in PDX1 expression across our microwell cultures correlated with the observed characteristic actin cytoskeletal architectures. Microwells with actin filaments concentrated at the center had significantly higher average PDX1 nuclear intensity (Fig. 5D) and PDX1 N:C ratio (not shown) than microwells with actin filaments concentrated at the periphery. This correlation suggests that cytoskeletal rearrangement of the micropatterned colonies is closely linked to the differentiation state of the cultures. Microwells hence bias cytoskeletal remodelling towards a clustered morphology, thereby improving differentiation efficiency as well as reducing differentiation variability.

To isolate the effect of cytoskeletal remodelling on confined, pancreatic differentiation, we added Y-26732, a selective inhibitor of ROCK^34^. Inhibition of the ROCK pathway in PF cells cultured in 150 µm microwells prevented actin reorganization and clustering (Fig. 5E). This also abrogated previously observed increases in mean PDX1 nuclear intensity compared to the surrounding unconfined PE cells. The Y-26732 treated microwells had similar cell numbers and densities to the untreated conditions, further suggesting that increases in PDX1 are dependent on cytoskeletal remodeling.

## Discussion

This work demonstrates that controlled clustering in adhesive microwells increases nuclear concentration of PDX1 and NKX6.1, two key transcription factors in beta cell development, in pancreatic endoderm (PE) cells. Inspired by spontaneous aggregation observed in standard unconfined monolayer cultures, we developed a simple, easy to handle, 2D system that enabled the reproducible generation of multicellular pancreatic clusters. The effect of confined culture was dependent on microwell size as well as the presence of soluble factors known to drive posterior foregut (PF) cells towards PE phenotypes. Confined culture was also correlated with distinct organizing behaviour of filamentous actin and clustering of cells within each microwell towards a multilayered, 3D, adherent microtissue phenotype. Although microwell-to-microwell variations exist, we observed a significant and robust increase in nuclear concentration of PDX1 and NKX6.1 in 150 μm diameter microwells compared to unconfined monolayer cultures.

The microwell-driven clustering method overcomes several key challenges in improving PSC differentiation towards mature pancreatic endocrine lineages. First, microwell cultures bias the stochastic formation of aggregates towards increased percentages of PDX1^high^ cells, compared to spontaneous aggregation in unconfined monolayer culture^3,8^. In 150 µm and 300 µm but not 500 µm microwells, increased N:C ratio of PDX1 was observed as well as clustering towards the center of the microwell; consistent with the approximate sizes of cell-assembled aggregates that occur in monolayer differentiation experiments. Our studies demonstrate that culturing differentiating cells in these patterns and at these length scales promotes the formation of actin architectures that encourage colonies to adopt a multilayered clustered morphology. The majority of microwell colonies that adopted this clustered phenotype were PDX1^high^ and also expressed higher levels of NKX6.1. Producing clusters of a defined size improves upon uncontrolled approaches such as coalescence in spinner flasks which results in aggregates with high size polydispersity. Since microenvironmental cues associated with aggregate size and density significantly impacts pancreatic differentiation^33,35^, the heterogeneity introduced during this aggregate formation step may explain the batch variability observed in beta cell yields, maturity, and purity between research groups.

Second, microwell cultures ensure highly repeatable cell densities across the substrates, which may significantly influence lineage specification, and is challenging to achieve with other aggregate-forming techniques that are amenable to culture for several days. This is particularly important to ensure uniform cell behaviour, which might be affected in complex ways. Compared to unconfined controls, increased PDX1 expression was observed in the 150 µm and 300 µm microwells, while a significant increase in NKX6.1 expression was only observed in the 150 µm microwells. This suggests that optimizing these processes for future production of PSC-derived beta cells will require consideration of a multiplexed panel of pancreatic biomarkers. The underlying reasons for these differences could be differences in cell density patterns between the microwell diameters that is known to influence PDX1 nuclear accumulation^8^, but also alters paracrine signalling gradients that are known to affect NKX6.1 expression, which requires a certain threshold of soluble factor stimulation^3^. Furthermore, the effect of microwell size on PDX1 and NKX6.1 expression suggests that random aggregation in unconfined monolayer cultures presents significant variability in endocrine differentiation. Since the microwell system produced PDX1^+^/NKX6.1^+^ co-positive clusters in a more controlled manner compared to those that formed randomly in conventional unconfined culture, microwell-based studies may ultimately provide fundamental insight into the driving factors behind lineage commitment.

Third, although the benefits of aggregation on pancreatic differentiation are well-established, conventional methods to form aggregates present handling challenges and require “forced” aggregation rather than spontaneous self-organized cluster formation as shown here. The throughput of the presented 2D micropatterning method is on a similar order as state-of-the art commercial aggregation methods. We estimate that around 1000 self-assembled, uniform, clusters could be produced per square centimeter of culture area, thus requiring roughly 600 cm^2^ to produce a therapeutic dose of ∼600,000 islet-like cell clusters per patient. Similarly, commercially available non-adhesive pyramidal microwells can produce roughly 700 similar sized clusters per square centimeter of culture area. However, harvesting requires repeated washing steps and aggregate recovery can vary from operator-to-operator^36^. Moreover, the commercially available pyramidal microwells, as well as other controlled suspension cell aggregation systems, do not allow self-organization of adherent cultures into multilayered microtissues as shown with the adhesive microwell system.

In this work we showed robust expression of elevated PDX1 and NKX6.1 nuclear accumulation in microwells at the PE stage of culture. Although other studies do report numerically higher frequencies of PDX1+ cells as early as the PF stage, we were unable to replicate these differentiation levels in unconfined cultures, perhaps due to others’ use of a lower threshold of PDX1 nuclear accumulation to define a positive cell^3,4,26,37^. Since our results demonstrate that levels of nuclear concentration of both PDX1 and NKX6.1 can be controlled based on the culture environment, and others have shown that levels of transcription factor expression can be important to improve downstream differentiation and function^38–40^, optimizing for the nuclear amount of these transcription factors may be a fruitful strategy, rather than optimizing for the number of positive or negative cells based on an arbitrarily defined threshold.

**Figure 6:**
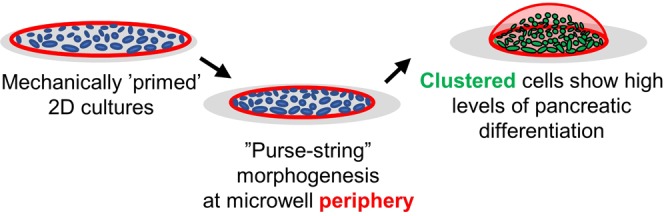
Proposed mechanism of action for increased PDX1 expression via collective actin “purse-string” contraction.

These findings do raise several questions about the stem cell differentiation process, particularly regarding the specific microenvironmental cues that drive enhanced lineage commitment at this stage in differentiation. Enhanced differentiation could be driven by many factor that arise in this culture model, including increased cell-to-cell contact, distinct soluble signal gradients within the microwell, mechanical stress gradients across the culture, the rearrangement dynamics of the actin cytoskeleton during aggregation, or simply allowing the microtissue to follow through on an intrinsic propensity to aggregate during differentiation^41,42^. Although the specific details of this transition remain open for investigation, our observations allow us to propose a putative model for enhanced pancreatic differentiation. Based on the progressive transition of F-actin patterning from uniform distribution to peripheral expression to concentration at the microwell center during the aggregation process, we believe that a ring of contractile actin drives “purse-string” morphogenesis^43^ to compact the aggregate structures (Fig. 6). The progressive contraction of the actin filaments from the microwell periphery correlates with higher levels of PDX1, suggesting that a dynamic culture process may prove to be an effective strategy to enhance differentiation during bioprocessing. While we hypothesize that clustering causes increased PDX1 expression, it is difficult to determine whether morphogenic changes lead to increased PDX1 expression or vice versa. Introducing approaches to downregulate PDX1 expression, such as siRNA, as well as time-lapse imaging studies with reporter PSC lines^3,44,45^ may help to understand the interplay between pancreatic differentiation and clustering. Further investigation into the dynamic relationship between NKX6.1 expression, actin reorganization, and further downstream beta cell development could inform bioprocessing strategies. Going further to develop this into a feasible bioprocessing strategy would require systematic optimization of microwell diameter, demonstration of scalability, and validation of beta cell functionality.

Overall, the presented microwell system is a simple, scalable and efficient method to produce self-organized pancreatic clusters in adherent cultures that could be used for patient-specific therapies or drug testing with cells that typically have poor pancreatic differentiation outcomes. Compared to unconfined adherent culture, the microwell system led to robust and consistent production of PDX1+/NKX6.1+ multilayered microtissues. Due to the increased accessibility of microfabrication techniques, fabrication of cell-adhesive microwells could be easily adapted into pancreatic differentiation protocols of other labs. The results from this work could inform the design and operation of new vessels for the scalable production of PE organoids by promoting cell-driven morphogenetic programs. By understanding cell behaviour during differentiation, novel substrates could be engineered to drive a larger percentage of cells towards pancreatic endocrine differentiation.

## Materials and Methods

### Cell culture and differentiation

Episomal iPSCs derived from CD34+ cord blood (Gibco, Cat # A18945) were maintained on Matrigel (Corning) coated plates in TeSR-E8 media (STEMCELL Technologies). For Matrigel coating, tissue culture treated 6-well plates were incubated with a Matrigel solution, diluted 1:25 in DMEM/F-12 medium (Fisher) for 1 h at room temperature. Media changes were performed every 24 h. Cells were passaged (1:6 dilution) with 0.5 mM EDTA in phosphate buffered saline solution (PBS) (Life Technologies) when reaching 70% confluency.

Prior to initiating differentiation, cells were cultured in mTeSR medium (STEMCELL Technologies) for 1 passage. To initiate differentiation, the iPSC cultures were dissociated with TrypLE Express (Life Technologies) and resuspended in mTeSR supplemented with 10 µM Y-26732 (ROCK inhibitor) (Sigma). The iPSCs were plated at a density of 200,000 cells/cm^2^ on Matrigel-coated plates. After 24 h, the adherent cells were rinsed with PBS and differentiation was initiated by the addition of differentiation medium. The differentiation medium was changed every 24 h according to the schedule shown in Table S3 which was based on a published protocol^5^. At the end of PF induction, the differentiated PF cells were frozen at a concentration of 1 million cells/mL in S3 media supplemented with 10% DMSO and frozen at a controlled −1 °C/min until −80 °C and then transferred to liquid nitrogen storage until required for confined culture experiments. Between each stage of differentiation, cells were washed with PBS.

### Quantitative polymerase chain reaction (qPCR)

Cell samples were collected as pellets (<5 million cells) and frozen at −20 °C for up to 2 weeks before RNA extraction. Extraction was performed using the QIAGEN RNeasy Mini kit according to the manufacturer’s protocol. Complementary DNA (cDNA) was produced with the High Capacity cDNA Reverse Transcription kit (Applied Biosystems) according to the manufacturer’s protocol. Next, qPCR was performed using the primers listed in Table S4 and Power SYBR Green Master Mix (Applied Biosystems). The samples were analyzed on an Applied Biosystems 7900HT Fast Real-Time PCR System. The thermal cycle used was: 50 °C for 2 min, 95 °C for 2 min, 40 cycles going from 94 °C for 30 s to 58  °C for 30 s to 72 °C for 30 s, 95 °C for 15 s, 60 °C for 15 s, 95 °C for 15 s, and 40 °C for 2 min.

### Immunocytochemistry (ICC)

Cell samples were fixed in 4% paraformaldehyde for 20 min, washed with PBS three times, permeabilized with 0.1% Triton-X100 solution for 20 min then washed with PBS three times. Non-specific protein adsorption was blocked by applying DAKO Protein Block Solution (Agilent) for 1 h at room temperature. After washing with PBS, the primary antibodies were incubated at 4 °C overnight, washed three times with PBS and then incubated with the corresponding secondary antibodies for 1 h at room temperature, and washed three times with PBS. Finally, samples were incubated with DAPI counterstain diluted in PBS for 20 min at room temperature and washed three times with PBS. The antibodies and dilutions used are list in Table S5.

### 2D geometric confinement using agarose microwells

Circular pillars were fabricated using SU-8 photolithography. Briefly, a 15 µm layer of SU-8 5 (MicroChem) was spin-coated at 4000 rpm for 1 min onto a sonicated, acetone-cleaned 50 mm × 75 mm glass slide. The slide was soft baked at 70 °C for 2 min and then hard baked at 100 °C for 5 min. The SU-8 was covered with the mask (CAD/ART Services, containing circular features as small as 50 µm) and exposed to UV light for 30 s in 5 s intervals to create a pattern of photo-crosslinked surfaces. The microfeatures were developed by gentle agitation in SU-8 developer. The resultant mold was baked at 100 °C for 5 min then flood exposed under UV for another 10 min.

These features were replicated using polydimethylsiloxane (PDMS) soft lithography to produce an inverse mold. An additional replication step was done in PDMS to produce stamps with pillars ranging from 150 µm to 500 µm in diameter. The PDMS stamps were plasma oxidized with atmospheric air for 2 min to create a hydrophilic surface and placed feature-side down on a glass slide. A hot solution of agarose and ethanol (3:2) was perfused through the pillars by capillary action by pipetting a small drop on the side of the stamp. The slides were then placed under vacuum for 1 h to allow gelation, after which the stamps were removed in a vertical motion using tweezers. Before seeding cells onto the substrate, the surface was coated with a layer of fibronectin by incubating in a 25 µg/mL solution for 1 to 2 h at room temperature. Frozen PF cells were seeded at 5.0 × 10^5^ cells/cm^2^ at day 7 out of 10 in the differentiation protocol to ensure confluency and to prevent tissue formation between micropatterns as the cells adhere. Cytoskeletal inhibition experiments were performed by adding 10 µM Y-26732 (ROCK inhibitor) for 48 hours starting from day 8 out of 10.

### Image analysis

The nuclear levels of transcription factors (PDX1 and NKX6.1) was quantified by image analysis using Fiji^46^. Nuclei were identified using the DAPI counterstain. Regions of interest were manually selected to encompass the nucleus (Fig. S7). The mean fluorescent intensity within the regions of interest was obtained as a measure of nuclear transcription factor concentration. Here, the nuclear cross-sectional area was assumed to be an adequate measure of nuclear volume due to the homogenous nuclei height across samples. To determine the nuclear-cytosolic ratio of PDX1 and NKX6.1 fluorescent intensity, the total cytosolic intensity of PDX1 and NKX6.1 was calculated by measuring the total transcription factor intensity encompassed by the tissue actin cytoskeleton (marked with fluorescent phalloidin staining) and subtracting the total nuclear fluorescent intensity. In unconfined samples, nuclei were chosen indiscriminately in both clustered and non-clustered areas. The surrounding cytoskeletal area of the confluent monolayer was selected to just encompass the nuclei selected for analysis. The nucleus-cytoplasmic (NC) ratio was then calculated as the mean nuclear fluorescent intensity divided by the mean cytoplasmic intensity.

The mean nuclear intensity of all cells measured within a microwell was then normalized to the intensity in the surrounding unconfined control (Fig. S8). This was done after background subtraction where the average background fluorescence of each image was obtained using the ‘Subtract Background’ algorithm in Fiji and subtracted from the raw fluorescent intensity measured. The radial position of a cell within the microwell was determined using x and y center of mass of the selected nuclei.

To confirm whether nuclear PDX1 fluorescent intensity of clustered cells is independent of any optical effects, we compared the fluorescent intensity of individual cells (n = 150) between: 1) spinning disc confocal microscopy and, 2) epifluorescent microscopy with a large depth of field. A constant ratio between the two imaging techniques in both clustered and non-clustered cells suggested changes in nuclear PDX1 fluorescent intensity was independent of optical effects.

Classification of actin morphology was done by qualitatively assessing the intensity profile of the phalloidin stain. The intensity profile was obtained by taking an average of the intensity profile across 8 lines, intersecting at the microwell center (Fig. S9). Microwells were classified as having an “peripheral” actin profile when the stain intensity was highest at the edge of the microwells and a “central” actin profile when the stain intensity was highest at the center of the microwell. The “uniform” morphology was determined when the actin stain covered the entire microwell and had a flat intensity profile.

### Statistical analysis

Statistical analysis was performed using JMP 14. Multiple comparisons were analysed using one-way ANOVA followed by Tukey multiple comparisons test while two-way comparisons were analysed using the Student’s t-test. In all analyses, p-values < 0.05 denote a statistically significant result. Three experimental replicates were performed for each experiment unless otherwise indicated in the figure captions.

### Ethics

Episomal iPSCs derived from CD34+ cord blood (Gibco, Cat # A18945) were maintained on Matrigel (Corning) coated plates in TeSR-E8 media (STEMCELL Technologies). All experiments conducted with pluripotent stem cell lines were approved by the Canadian Stem Cell Oversight Committee.

## Supplementary information

Supplementary data.

## Data Availability

The data supporting the findings of this study are available within the paper and its Supplementary Information.
