# Supplementary material for: Controlled clustering enhances PDX1 and NKX6.1 expression in pancreatic endoderm cells derived from pluripotent stem cells
Source: Sci Rep. 2020 Jan 27;10:1190. doi: 10.1038/s41598-020-57787-0 (PMC6985188; doi:10.1038/s41598-020-57787-0)
Supplement: Supplementary file 1 — Supplementary data. [file 41598_2020_57787_MOESM1_ESM.docx]

# Supplemental Information

**Controlled clustering enhances PDX1 and NKX6.1 expression in pancreatic endoderm cells derived from pluripotent stem cells**

Raymond Tran^1^, Christopher Moraes^1,2,3^*, Corinne A. Hoesli^1^*

^1^ Department of Chemical Engineering, McGill University, 3610 rue University, Montreal, QC, Canada

^2^ Department of Biomedical Engineering, McGill University, 3775 rue University, Montreal, QC, Canada

^3^ Rosalind and Morris Goodman Cancer Research Center, McGill University, Montreal, QC, Canada

*Corresponding authors & equal contribution

E-mail: [chris.moraes@mcgill.ca](mailto:chris.moraes@mcgill.ca); [corinne.hoesli@mcgill.ca](mailto:corinne.hoesli@mcgill.ca)

**Table S1:** Significance testing summary for one-way ANOVA with Tukey post-hoc multiple comparison on qPCR data presented in Figure 1B. n.s.=p>0.05, *=p<0.05, **=p<0.01, ****=p<0.001.

|  | **OCT3/4** | **SOX17** | **FOXA2** | **HNF6** | **PDX1** | **NKX2.2** | **NKX6.1** |
| --- | --- | --- | --- | --- | --- | --- | --- |
| **Overall Significance** | ******** | ***** | **n.s.** | ****** | ****** | ****** | **n.s.** |
| iPSCs vs DE | **** | * | n.s. | n.s. | n.s. | n.s. | n.s. |
| iPSCs vs PF | **** | n.s. | n.s. | n.s. | n.s. | n.s. | n.s. |
| iPSCs vs PE | **** | n.s. | n.s. | ** | * | ** | n.s. |
| DE vs PF | n.s. | n.s. | n.s. | n.s. | n.s. | n.s. | n.s. |
| DE vs PE | n.s. | * | n.s. | ** | * | * | n.s. |
| PF vs PE | n.s. | n.s. | n.s. | * | n.s. | ** | n.s. |

**Table S2:** Significance testing summary for one-way ANOVA with Tukey post-hoc multiple comparison on cell density data presented in Figure 2F. n.s.=p>0.05, **=p<0.01, ****=p<0.001.

|  | **Microwell Diameter (µm)** | | |
| --- | --- | --- | --- |
|  | *150* | *300* | *500* |
| **Overall Significance** | ******** | ******** | ******** |
| Region 1 vs Region 2 | ** | n.s. | ** |
| Region 1 vs Region 3 | **** | **** | n.s. |
| Region 1 vs Region 4 | **** | **** | **** |
| Region 2 vs Region 3 | **** | **** | **** |
| Region 2 vs Region 4 | **** | **** | **** |
| Region 3 vs Region 4 | **** | **** | **** |

**Table S3:** Detailed composition of differentiation medium used in each stage to produce PF cells.

| **Days** | **Medium** | **Basal Medium** | **Soluble Factors** |
| --- | --- | --- | --- |
| 1 | S1A | MCDB131 + 10mM glucose + 1.5 g/L NaHCO_3_ + 0.5% fatty acid free bovine serum albumin (FAF-BSA) + 1x GlutaMAX + 1% Pen/Strep | +100 ng/mL Activin A +3 μM CHIR99021 |
| 2-3 | S1B |  | +100 ng/mL Activin A |
| 4-5 | S2 |  | +0.25 mM ascorbic acid +50 ng/mL keratinocyte growth factor (KGF) |
| 6-7 | S3 | MCDB131 + 10mM glucose + 2.5 g/L NaHCO_3_ + 2% FAF-BSA + 1x GlutaMAX + 1% Pen/Strep | +0.25 mM ascorbic acid + 1:200 insulin-transferrin-selenium-ethanolamine (ITS-X) +50 ng/mL KGF + 0.25 µM SANT-1  + 1 µM retinoic acid  + 100 nM LDN193189  + 200 nM TPB (PKC activator) |
| 8-10 | S4 |  | +0.25 mM ascorbic acid + 1:200 ITS-X  +2 ng/mL KGF + 0.25 µM SANT-1  + 0.1 µM retinoic acid  + 200 nM LDN193189  + 100 nM TPB |

**Table S4:** Primers used for qPCR

| **Gene** | **Forward / Reverse Primer** | **Sequence (5'-3')** |
| --- | --- | --- |
| GAPDH | Forward | CCCATCACCATCTTCCAAGGAG |
|  | Reverse | CTTCTCCATGGTGGTGAAGACG |
| OCT 3/4 | Forward | TGGGCTCGAGAAGGATGTG |
|  | Reverse | GCATAGTCGCTGCTTGATCG |
| SOX2 | Forward | CACAACTCGGAGATCAGCAA |
|  | Reverse | TCCGGGAAGCGTGTACTTA |
| SOX17 | Forward | GGCGCAGCAGAATCCAGA |
|  | Reverse | CCACGACTTGCCCAGCAT |
| FOXA2 | Forward | GGGAGCGGTGAAGATGGA |
|  | Reverse | TCATGTTGCTCACGGAGGAGTA |
| HNF1B | Forward | TCACAGATACCAGCAGCATCAGT |
|  | Reverse | GGGCATCACCAGGCTTGTA |
| HNF4A | Forward | CATGGCCAAGATTGACAACCT |
|  | Reverse | TTCCCATATGTTCCTGCATCAG |
| HNF6 | Forward | CGCTCCGCTTAGCAGCAT |
|  | Reverse | GTGTTGCCTCTATCCTTCCCAT |
| PDX1 | Forward | AAGTCTACCAAAGCTCACGCG |
|  | Reverse | GTAGGCGCCGCCTGC |
| NKX2.2 | Forward | CCGAGGGCCTTCAGTACTCC |
|  | Reverse | CGGGGTCTCCTTGTCATTGT |
| NKX6.1 | Forward | TTCGCCCTGGAGAAGACTTT |
|  | Reverse | GCGTGCTTCTTCCTCCACTT |

**Table S5**: Antibodies and reagents used for immunocytochemistry

| **Antibody** | **Supplier** | **Catalog #** | **Dilution** |
| --- | --- | --- | --- |
| Rabbit anti-human mAb HNF4α | Abcam | ab92378 | 1:400 |
| Mouse anti-human mAb PDX1 | BD Pharmigen | 562160 | 1:200 |
| Rabbit anti-human pAb NKX6.1 | Novus Biologicals | NBP149672 | 1:200 |
| Goat anti-mouse AlexaFluor 488 | Life Technologies | A11001 | Primary |
| Goat anti-rabbit AlexaFluor 568 | Life Technologies | A11011 | Primary |
| 4′,6-Diamidino-2-phenylindole dihydrochloride (DAPI) | Life Technologies | D9542 | 1:1000 |
| TRITC-conjugated phalloidin | Life Technologies | P1951 | 1:200 |


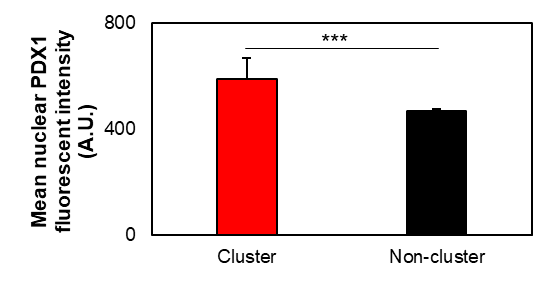


**Figure S1*:*** Clustered (n = 7) pancreatic endoderm cells have elevated nuclear PDX1 fluorescent intensity than surrounding non-clustered cells. ***=p<0.005 for a Student’s t-test.


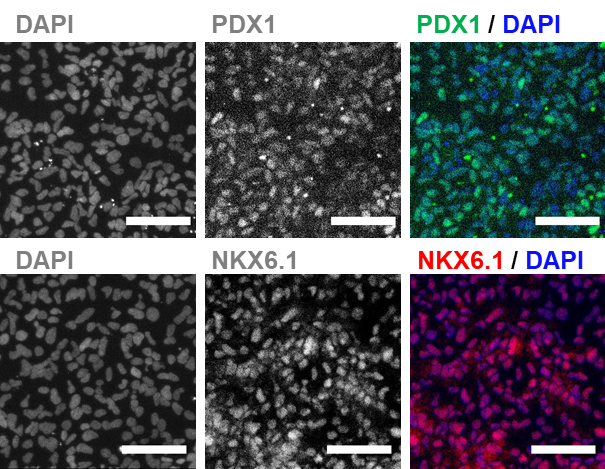


**Figure S2:** Unconfined pancreatic endoderm cultures show positive staining for pancreatic transcription factors PDX1 and NKX6.1. Brightness and contrast have been increased to show positive staining. Scale bars: 100 µm.


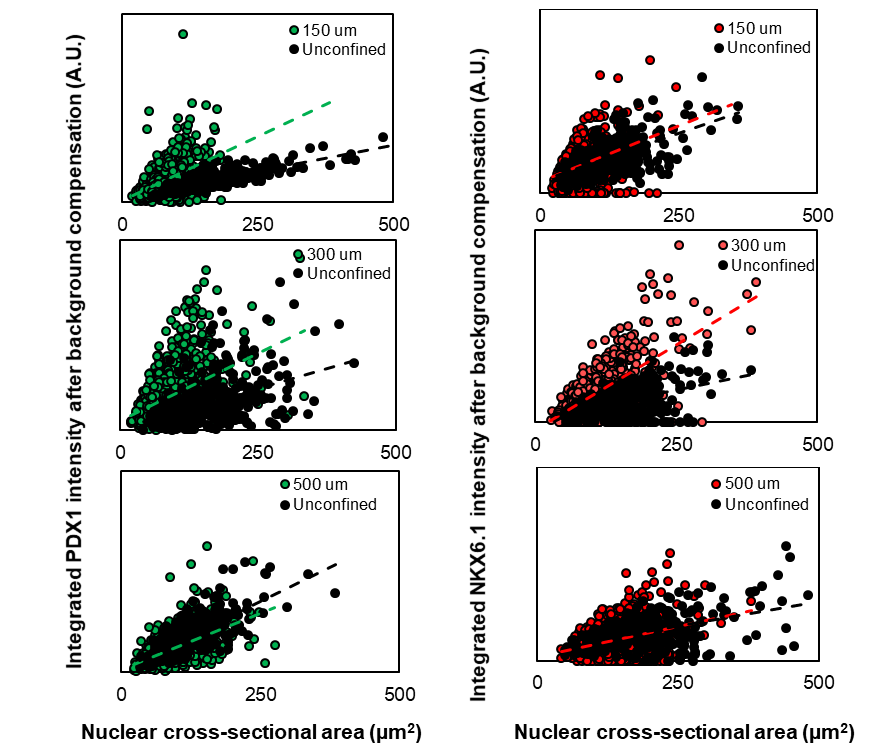


**Figure S3:** Representative plots of total nuclear fluorescence intensity. Each point represents a single nucleus within each culture condition. The effects of nuclear area and confined culture were determined by applying the following linear model: y = β_0_ + β_1_x_1_ + β_2_x_2_, where y is the total nuclear fluorescence intensity, x_1_ is the culture system and x_2_ is the nuclear cross-sectional area. The nuclear area had a statistically significant positive correlation with the integrated fluorescence intensity of PDX1 or NKX6.1 staining in all conditions (p<0.0001). Confined culture had a significant positive effect on PDX1 and NKX6.1 integrated intensity in the 150 µm and the 300 µm diameter microwells, while a small but significant (p<0.02) negative effect was observed for 500 µm microwell confined culture compared to unconfined controls.


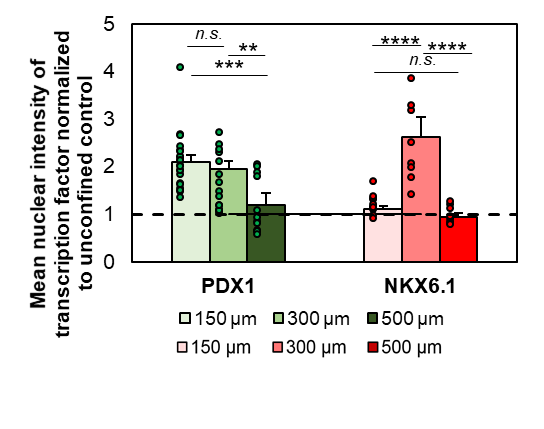


**Figure S4:** PDX1 (n = 21, 13, 9 for 150, 300, and 500 µm microwells) and NKX6.1 (n = 12, 8, 8 for 150, 300, and 500 µm microwells) nuclear intensity is increased when presented with sufficient geometric confinement. Each point represents a data point from a single microwell. n.s = p>0.05, **=p<0.01, ***=p<0.005, ****=p<0.001.


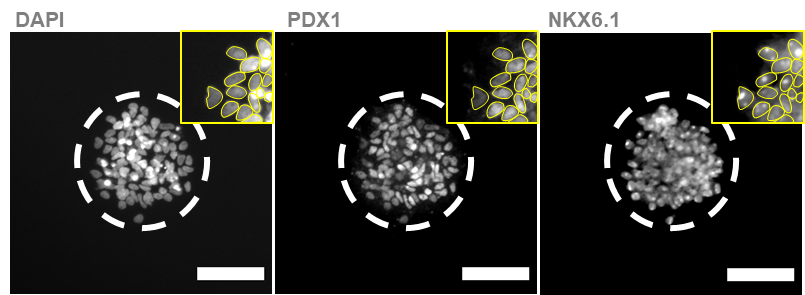


**Figure S5:** Double stained 150 µm microwells show nuclear colocalization of PDX1 and NKX6.1. Scale bars: 100 µm.


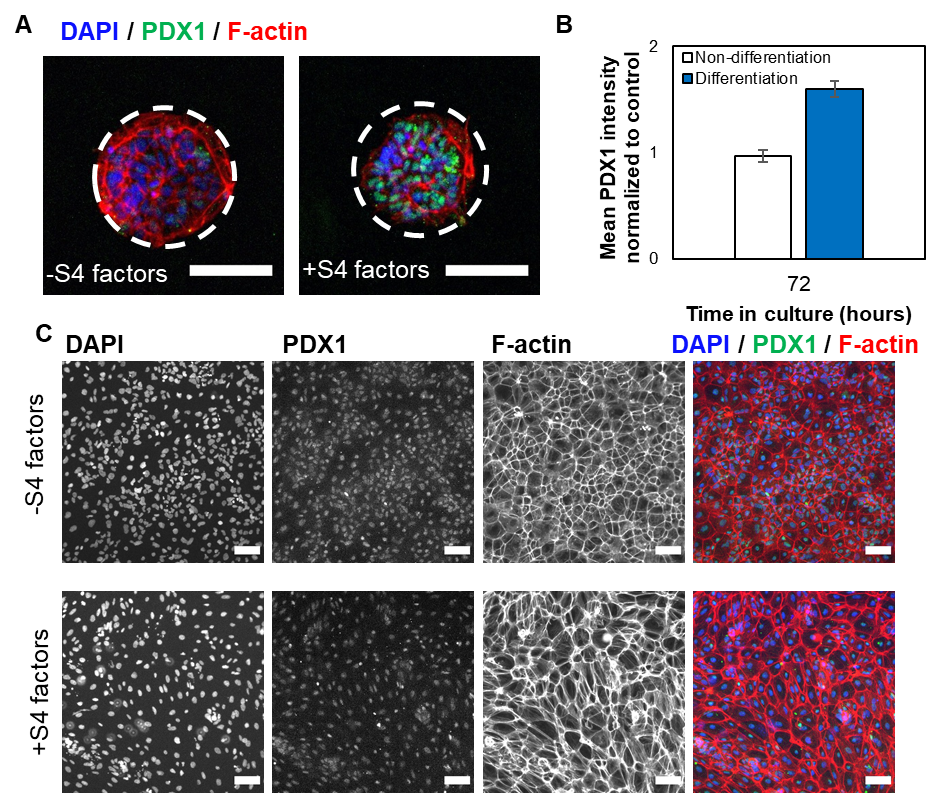


**Figure S6**: Culture in absence of differentiation inducing factors abrogates increased PDX1 expression, reduces cell density, and disrupts benefits of microwell culture. (A) Removal of S4 differentiation factors halts cytoskeletal reorganization and abrogates any improvements in pancreatic differentiation from microwell culture shown by immunocytochemistry of PF cells confined within 150 µm wells. (B) Confined culture in absence of soluble factors does not upregulate PDX1 expression over the unconfined control. (C) Decreased cell density is observed in unconfined controls when PE inducing differentiation factors are removed. Scale bars: 100 µm.

*
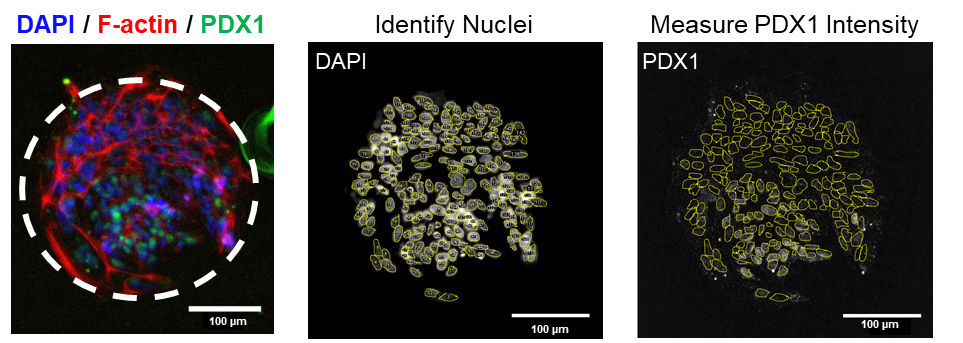
*

**Figure S7**: Image analysis work flow. First nuclei were manually selected as regions of interest using the DAPI counterstain. Next, the intensity of the PDX1 stain was measured within the selected regions of interest.


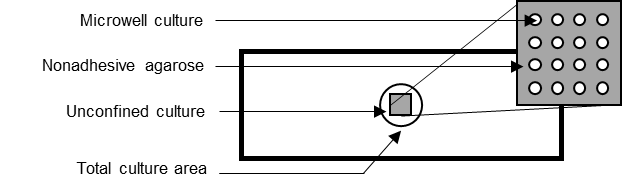


**Figure S8:** Layout of slide containing microwell cultures.


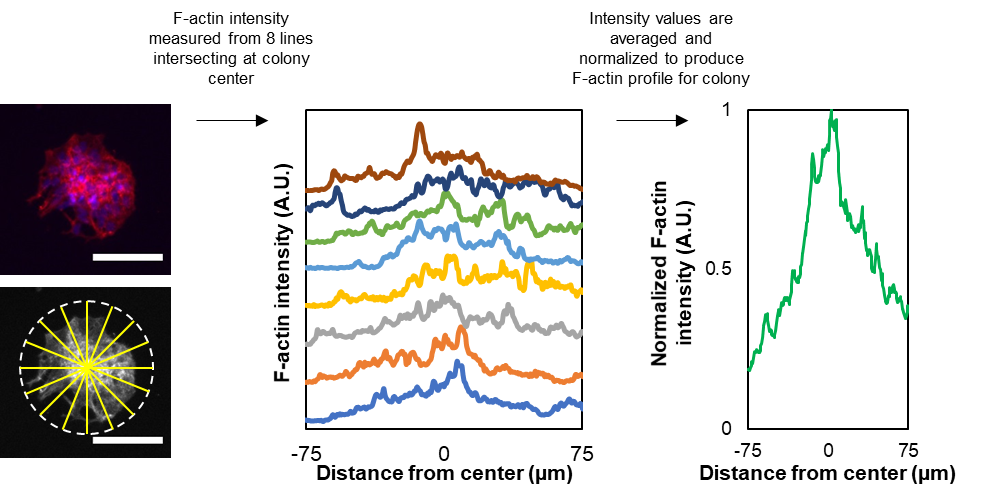


**Figure S9**: Process flow to obtain actin intensity profile shown in Figure 5B. The reported actin intensity profiles are the average of 8 actin profiles which intersect at the microwell colony center.
